# Supplementary material for: Two-dimensional flow nanometry of biological nanoparticles for accurate determination of their size and emission intensity
Source: Nat Commun. 2016 Sep 23;7:12956. doi: 10.1038/ncomms12956 (PMC5036154; doi:10.1038/ncomms12956)
Supplement: Supplementary Information — Supplementary Figures 1-11, Supplementary Notes 1-7 and Supplementary References [file ncomms12956-s1.pdf]

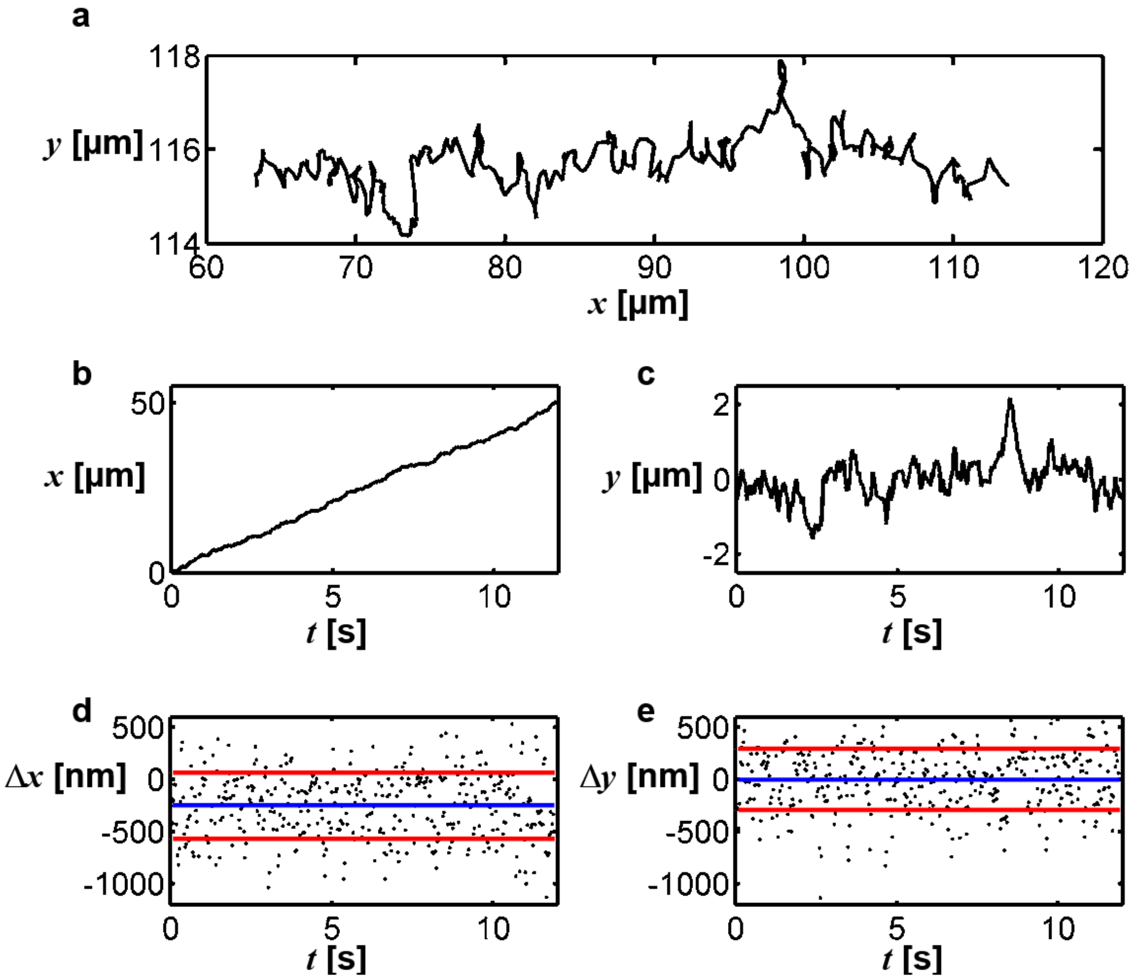

Supplementary Figure 1: **Extraction of stochastic and deterministic components of NP movement.** Representative trajectory (**a**) of a single gold NP (hydrodynamic radius 30 nm; channel flow rate 15 µL min^{-1}), and the corresponding decomposition into the x- and y-component of the movement (**b**, **c**), and the x- and y-component of the particle displacement (**d**, **e**) for a lag of 2 frames (*i.e.*, $\Delta x(i) = x(i{+}2) - x(i)$; see text for details). The lines in **d** and **e** give average value (blue line) plus/minus standard deviation (red lines) of the calculated displacement values. Due to the flow, the average value (blue line) is non-zero in **d**, indicative for the directed movement (see also the almost linear increase in **b**), which is superimposed by a random movement (1D diffusion; see fluctuations in **b** and **d**). Perpendicular to the flow (**c**, **e**), the particle shows only the random movement and the average displacement vanishes. In both cases, the standard deviation is proportional to the square root of the diffusion coefficient in the respective direction, which allows to extract D_x and D_y from the decomposed data.

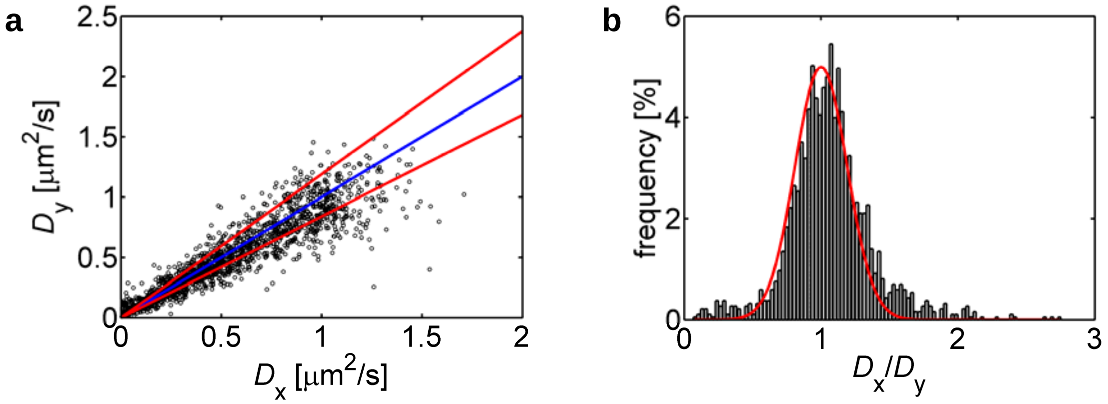

Supplementary Figure 2: **Comparison of extracted 1D diffusion coefficients D_x and D_y.** Shown are a parameter plot of D_x versus D_y (**a**) and the normalized probability distribution of the ratio D_x / D_y (**b**) of gold NPs (hydrodynamic radius 30 nm). The blue line in (**a**) indicates the expected 1:1 relation, while the red lines give the boundaries of the error intervals as expected from Eq. 16. As the SLB is isotropic, D_x and D_y are expected to be identical and follow the 1:1 relation (blue line) in absence of measurement noise. However, due to measurement noise both diffusion coefficients fluctuate with a standard deviation given by Supplementary Equation 16 around their real value, which is indicated here by the fact that the majority of the data points are located within the boundaries of the error intervals (red lines). Hence, the observed deviation from the 1:1 relation is mainly caused by the statistically expected measurement noise, and not by errors done in the data analysis. This is also reflected by the normalized probability distribution of the ratio D_x / D_y (**b**; bars), which is well described by a Gaussian distribution (**b**; red line) using the standard deviation given by Supplementary Equation 16.

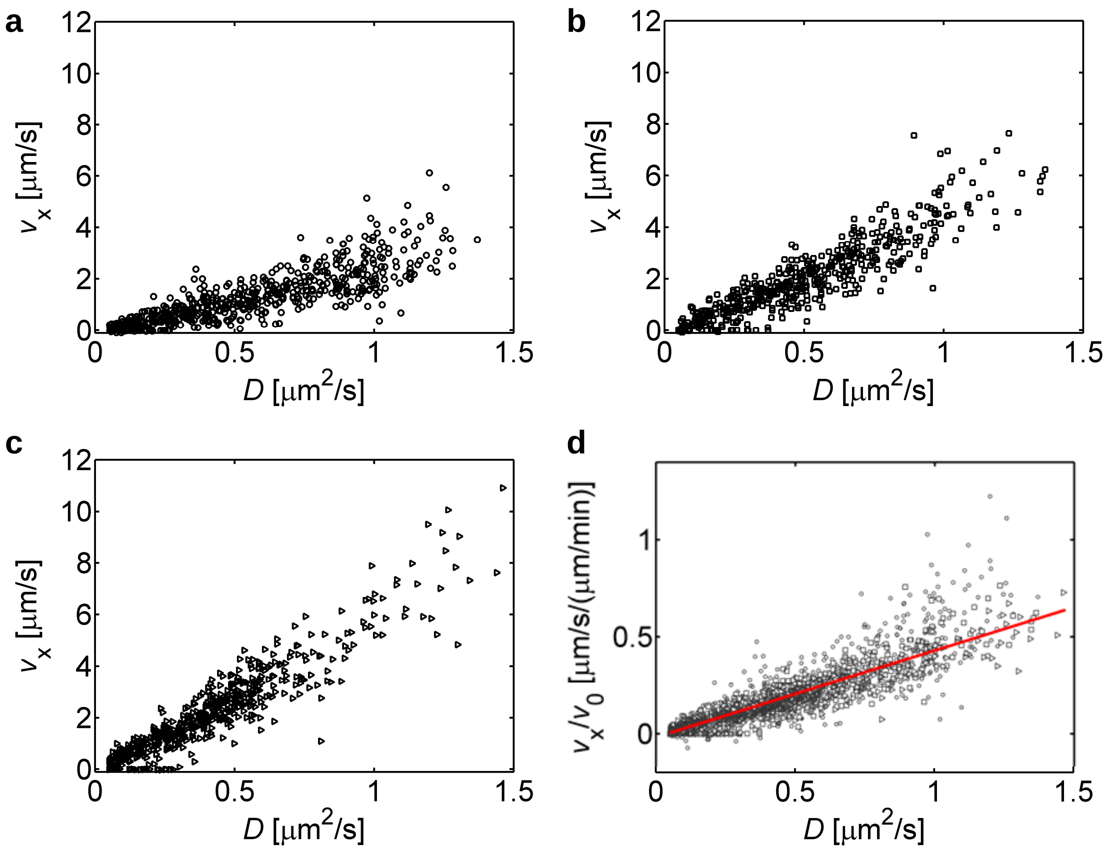

Supplementary Figure 3: **Normalization of NP drift rate by flow rate.** Velocity v_x induced for gold NPs (hydrodynamic radius 30 nm) by flow rates of 5 μL min^{-1} (**a**), 10 μL min^{-1} (**b**), and 15 μL min^{-1} (**c**) versus NP diffusion coefficient D (defined as average of D_x and D_y). The raw data (**a-c**) shows an increase in velocity with increasing diffusion coefficient D and flow rate, while all data point collapse onto a single master curve (red line) after normalization of the NP velocity by the flow rate (**d**).

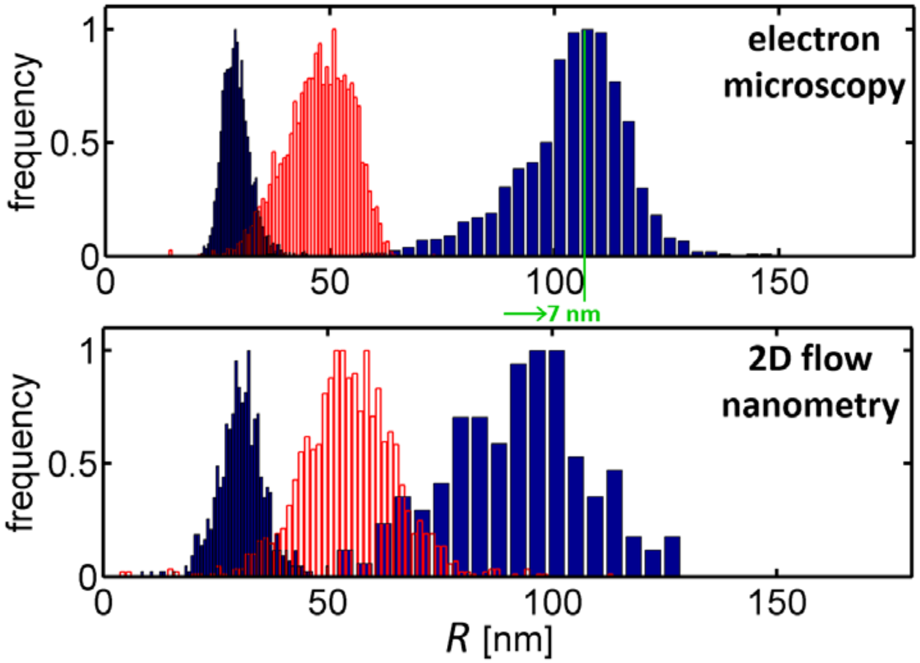

Supplementary Figure 4: **2D flow nanometry versus electron microscopy.** The corresponding NP size distributions are shown for three different batches of gold NPs.

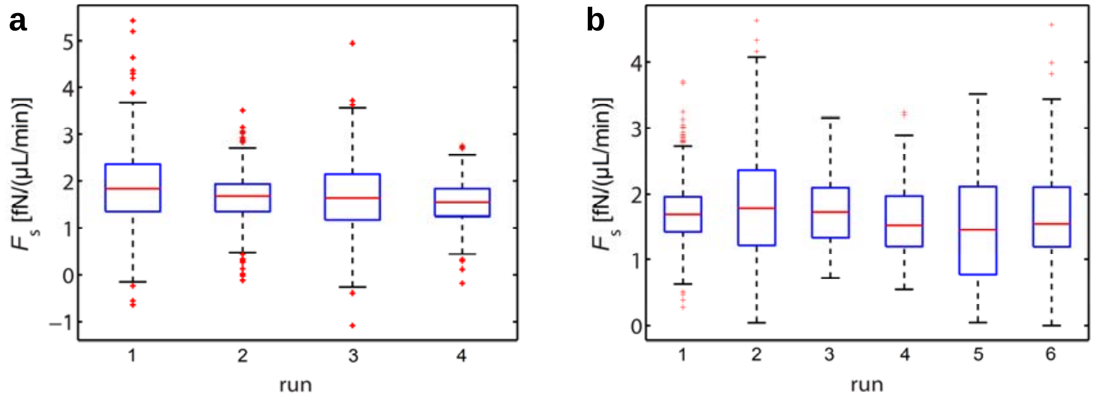

Supplementary Figure 5: **Reproducibility in hydrodynamic force extraction.** Boxplots of hydrodynamic forces (normalized by the flow rate) extracted from several 2D flow nanometry measurements of gold NPs (30 nm hydrodynamic radius). In (**a**) the NPs have been functionalized with streptavidin and linked to biotinylated lipids in the SLB, while in (**b**) the NPs have been functionalized with antibodies and specifically bound to BACE1 transmembrane proteins incorporated in the SLB (as recently described[1]). **Run 1** and **3** in (**a**) were done using a flow rate of 5 μL min^{-1}, while **run 2** and **4** in (**a**) and **all runs** in (**b**) used 10 μL min^{-1}. Irrespective of the linking strategy, the peak positions (red lines) of the normalized hydrodynamic force fluctuate less than 0.2 fN min $μL^{-1}$ around the average value of 1.6 fN min $μL^{-1}$, indicating high reproducibility.

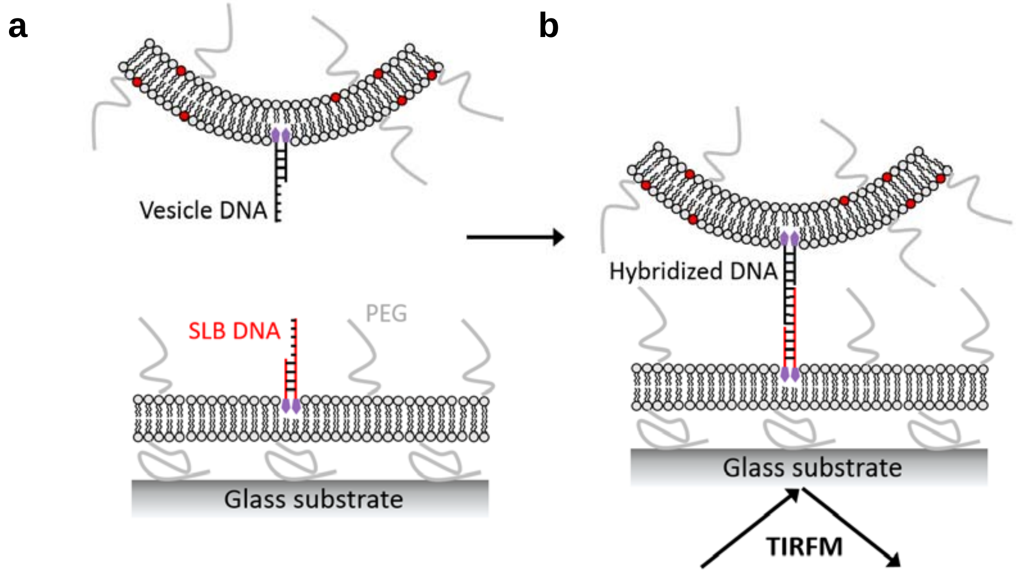

Supplementary Figure 6: **DNA-mediated vesicle linking to a SLB used for single particle tracking (SPT).** Vesicle and SLB DNA (black and red strands, respectively) carry a double cholesterol group at their ends that insert the strands into bilayers. SLB DNA is only pre-incubated with the SLB, while vesicle DNA is only pre-incubated with vesicles (**a**). Mixing of DNA-equipped vesicles with a DNA-equipped SLB causes hybridization, thereby linking the vesicles via DNA-tethers to the SLB (**b**; showing only a single linking DNA-tether for simplicity). PEGylated lipids have been incorporated to decouple the lipid motion from surface effects and to decrease non-specific attachment to the SLB.

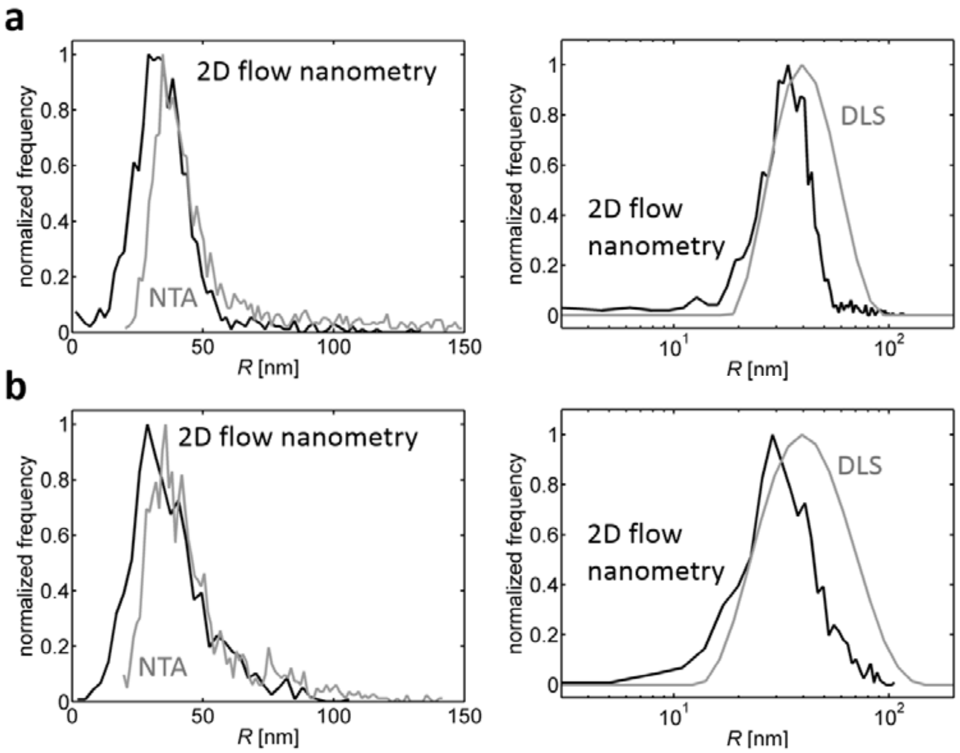

Supplementary Figure 7: **2D flow nanometry versus NTA, and DLS.** Vesicle size distributions of 2 different batches, prepared using extrusion (**a**) and sonication (**b**) as described in the Methods section in the main text, obtained using fluorescent NTA (**left column**), DLS (**right column;** intensity distributions) and 2D flow nanometry.

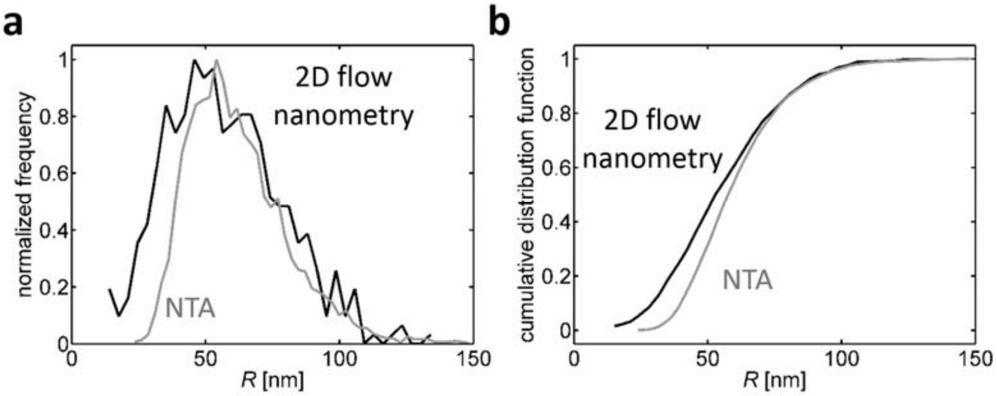

Supplementary Figure 8: **2D flow nanometry versus NTA.** Vesicle size distributions (**a**) and the corresponding cumulative distribution functions (**b**) for an additional batch (with somewhat larger average size), prepared using extrusion as described in the Methods section in the main text (but employing 100 nm pores) obtained using fluorescent NTA and 2D flow nanometry.

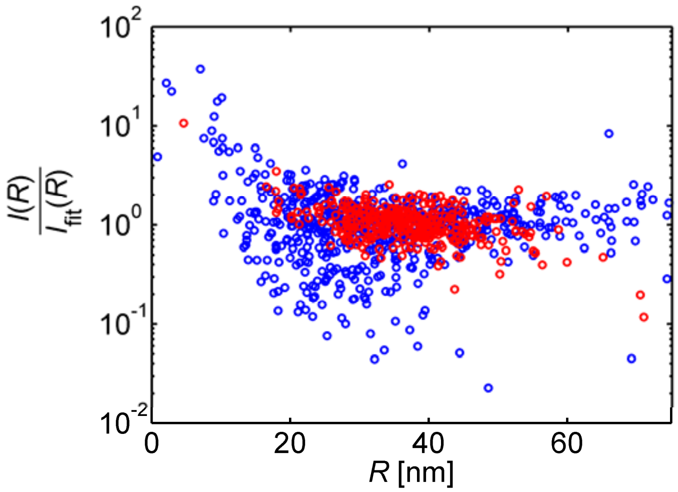

Supplementary Figure 9: **Degree of inhomogeneity of small unilamellar vesicles.** Ratio between measured ($I(R)$) and fitted vesicle intensity ($I_{fit}(R)$, solid lines in Fig. 5c, d in the main text), which was used by Larsen et al.[2] to assess heterogeneities in lipid composition of individual vesicles (blue dots = Figure 5c; red dots = Figure 5d). The standard deviation of this ratio, which is equivalent to the degree of inhomogeneity as introduced by Larsen et al.[2], is 0.41 (blue dots) and 0.30 (red dots), respectively.

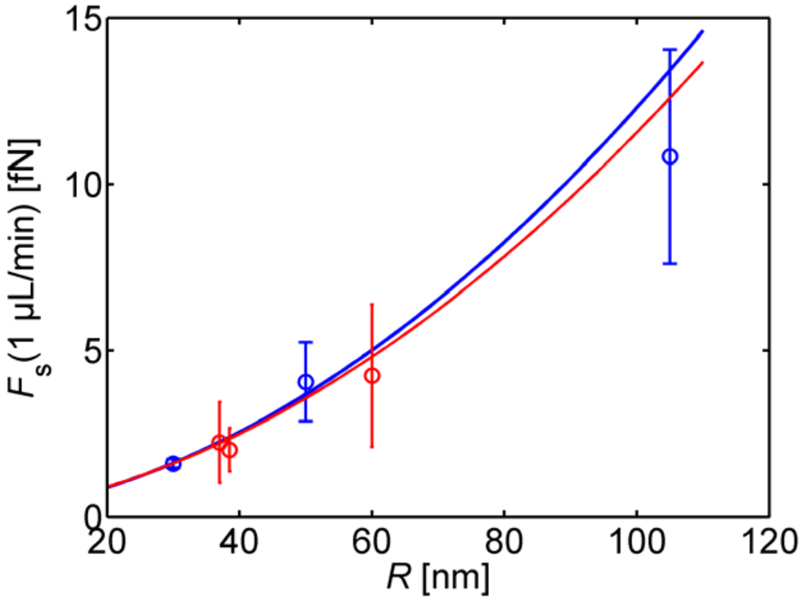

Supplementary Figure 10: **Calibration of 2D flow nanometry using gold NP and vesicle datasets.** Relationship of hydrodynamic force (normalized by the flow rate) versus NP size determined from 2D flow nanometry on gold NPs (blue circles; flow rates of 5, 2.5 and 1 µL min^{-1} for R = 30, 50 and 105 nm, respectively) and vesicles (red circles; flow rate of 5 µL min^{-1}) and fits to Supplementary Equation 12 using only gold NP data (blue line) and all data points (red line).

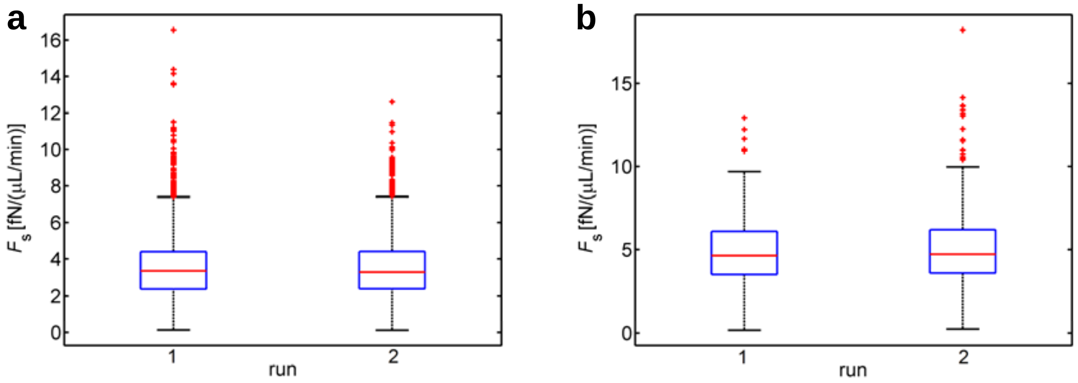

Supplementary Figure 11: **Illustration of the absence of flow-induced vesicle deformation.** Boxplots of hydrodynamic forces (normalized by the flow rate) extracted from several 2D flow nanometry measurements of 2 vesicle batches (**a**) and (**b**). Run 1 represents in both cases a flow rate of 2.5 µL min^{-1}, while run 2 corresponds to 5 µL min^{-1}.

Supplementary Note 1

Accuracy of SPT-based size extraction - All single particle tracking (SPT) implementations reported so far to determine the nanoparticle (NP) size (*e.g.*, the so-called NP tracking analysis, NTA), record the NP movement in a bulk solution, extract the bulk diffusion coefficient D_b from the recorded trajectory and use the Stokes-Einstein relation between D_b and the hydrodynamic radius, R, of the tracked NP,

$$D_{\mathrm{b}} = \frac{k_{\mathrm{B}} \cdot T}{6\pi \cdot \eta \cdot R}, \qquad (1)$$

where k_B denotes the Boltzmann constant, T the absolute temperature, and η the dynamic viscosity.[3] Among the experimentally measured parameters entering Supplementary Equation 1, the determination of D_b causes the largest random errors, which is a consequence of the intrinsic noise of the underlying random walk process.[4, 5] Hence, to estimate the random error σ_R in the determination of R (expressed here by the standard deviation of the measured R for NPs having an identical size, $\sigma_R = \langle \Delta R^2 \rangle^{1/2}$), it is sufficient to only regard the random error σ_{D_b} (in the determination of D_b). It is obvious from Supplementary Equation 1 that the relative errors σ_R / R and σ_{D_b} / D_b are equal,

$$\frac{\sigma_R}{R} = \frac{\langle \Delta R^2 \rangle^{1/2}}{R} = \frac{\langle \Delta D_{\mathrm{b}}{}^2 \rangle^{1/2}}{D_{\mathrm{b}}} = \frac{\sigma_{D_{\mathrm{b}}}}{D_{\mathrm{b}}}. \qquad (2)$$

The diffusion coefficient of a particular two-dimensional (2D) trajectory is usually determined using the approach of *internal averaging*,[4, 5] in which the squared displacements of all particle positions being separated by a given lag time τ are calculated, followed by averaging of these squared displacements. For a 2D random walk, this mean squared displacement, MSD, increases linearly with τ according to $\mathrm{MSD} = 4D_{\mathrm{b}}\tau$, allowing to extract D_b from a linear fit of the obtained MSD-τ curve. For a trajectory covering N frames, the extracted D_b value is well known to fluctuate around the true value with a relative standard deviation of

$$\frac{\sigma_{D_{\mathrm{b}}}}{D_{\mathrm{b}}} = \frac{\langle \Delta D_{\mathrm{b}}{}^2 \rangle^{1/2}}{D_{\mathrm{b}}} = \sqrt{\frac{2}{3} \cdot \frac{N_{\mathrm{p}}}{N - N_{\mathrm{p}}}} \qquad \text{(for } N_{\mathrm{p}} \ll N\text{)} \qquad (3)$$

where N_p is the maximum data point separation used in the internal averaging (i.e., N_p is the maximum lag time involved in the extraction of D_b divided by the time between 2 consecutive frames, Δt_0: $N_{\mathrm{p}} = \max(\tau)/\Delta t_0$).[4, 5] Hence, high accuracy in the determination of R requires high accuracy in the determination of D_b and therefore trajectories containing as much data points as possible (*i.e.*, extending over as many frames N as possible). Note that Supplementary Equation 3 does not contain any

contribution from the localization noise,[6] as the acquisition rate $1/\Delta t_0$ can usually be chosen small enough so that the stochastic noise apprecially exceeds the localization noise.

Due to the finite depth of focus, z_R, objects can only be tracked for a certain period of time, the average value of which, Δt, can be estimated based on D_b

$$\Delta t = z_R^2 / 2D_b . \tag{4}$$

Hence, to increase the measurement accuracy, the length of the trajectories must be increased, which is solved in this work by linking the NPs to an interface and by determining the hydrodynamic shear force F_s that acts on the NPs due to the action of a well-defined shear flow. In this case, the random error σ_{F_s} in the determination of F_s can be expressed via the errors σ_D and σ_{v_x} in the determination of the 2D diffusion coefficient, D, and the flow-induced velocity, v_x, as

$$\left(\frac{\sigma_{F_s}}{F_s}\right)^2 = \left(\frac{\sigma_{v_x}}{v_x}\right)^2 + \left(\frac{\sigma_D}{D}\right)^2 . \tag{5}$$

The ratio σ_D / D is again given by Supplementary Equation 3,[4, 5]

$$\frac{\sigma_D}{D} = \sqrt{\frac{2}{3} \cdot \frac{N_p}{N - N_p}} , \qquad \text{(for } N_p \ll N\text{)} \tag{6}$$

while the error σ_{v_x} can be calculated based on the equations for the standard error of a linear regression:[7]

$$\sigma_{v_x} = \sqrt{\frac{24 \cdot D}{\Delta t_0} \cdot \frac{N_p}{\left(N - N_p\right)^3}} . \qquad \text{(for } N_p \ll N\text{)} \tag{7}$$

Inserting typical values (D ~ 1 µm² s^{-1}, Δt_0 ~ 0.1/s, N_p ~ 2, N > 100) shows that Supplementary Equation 5 is dominated by the error σ_D for induced velocities v_x exceeding 1 µm s^{-1}, which are experimentally easy to achieve by adjusting the flow rate through the channel.

To relate Supplementary Equation 5 above with σ_R in the size determination by using Eq. 5 (in the main text), we take into account that at $R \gg \lambda$ the latter equation can be approximated as

$$F_s(R) \propto R \cdot (R + \lambda) \approx R^2 \tag{8}$$

yielding

$$\sigma_R / R = \sigma_{F_s} / 2F_s . \qquad \text{(for } R \gg \lambda\text{)} \tag{9}$$

and therefore

$$\frac{\sigma_R}{R} = \frac{1}{2}\cdot\sqrt{\frac{2}{3}\cdot\frac{N_\mathrm{p}}{N-N_\mathrm{p}}}\,. \qquad \text{(for } N_\mathrm{p} \ll N \text{ and } R \gg \lambda\text{)} \tag{10}$$

This is half of the error given by Supplementary Equation 3. Hence, for trajectories having the same length N, the measurement accuracy is already improved after linking (with respect to the case of bulk diffusion) due to the non-linear relationship between the hydrodynamic radius and hydrodynamic shear force. Moreover, the accuracy can be further improved after linking as (in contrast to Supplementary Equation 4) the transition time of the objects through the field of view is determined by the induced velocity v_x, which can be controlled by the applied flow rate. Hence, depending on the size distribution (that has to be measured), the flow rate can be adjusted to yield an induced velocity v_x that is on one hand large enough so that the σ_{v_x} contribution is negligible in Supplementary Equation 5, but on the other hand is still small enough to maximize the transition time and therefore the length N of the trajectories. This adjustment possibility is lacking in bulk-based methods and a hallmark of 2D flow nanometry.

Supplementary Note 2

Hydrodynamic shear force above a lipid bilayer - The channel height of common microfluidic channels is usually much larger than the NP hydrodynamic sizes of interest. For low Reynolds numbers (laminar flow) and the conventional ”no-slip” boundary condition, this allows to approximate the parabolic flow profile by $v_\mathrm{fluid}(z) = u_0 \cdot z$, where u_0 is the gradient of the velocity at the interface, and z is the coordinate perpendicular to the interface. In fact, this expression for v_fluid is based not only on the ”no-slip” boundary condition but also on the assumption that the conventional hydrodynamics holds down to z = 0, or, more specifically, that the viscosity coefficient is constant down to z = 0. Both these conditions are widely accepted in the literature. In our context, for example, we may note that the solution dynamics near an SLB under QCM-D conditions is successfully described by using the ”no-slip” boundary condition.[8] Another relevant example is that the experimentally measured slowdown of diffusion of NPs near the surfaces can be described by employing the conventional theory (see *e.g.* Kazoe et al.[9] and references therein). On the other hand, there are recent reports[10-14] indicating that just near the interface the applicability of the conventional hydrodynamics with the constant viscosity coefficient and ”no-slip” boundary condition is limited and that the flow profile can there be represented as

$$v_\mathrm{fluid}(z) = u_0 \cdot (z+\lambda) \tag{11}$$

where the length λ may be on the scale of 10 nm for SLB.[11, 13] In the literature λ is often referred to as ”slip length”. In fact, however, this is rather the length of the region where the convenitional hydrodynamics is expected to be modified.

For laminar flows, the gradient in Supplementary Equation 11 scales linearly with the flow rate v_0, *i.e.*, $u_0 \propto v_0$. Further, a scaling analysis shows that the drag force acting on a spherical particle in such a laminar flow scales with the product of flow velocity at $z = R$ (the middle of the NP) times the hydrodynamic radius R,[15-17] allowing us to represent the drag force as

$$F_s(R) = A \cdot \eta \cdot v_0 \cdot R \cdot (R + \lambda) , \qquad (12)$$

with A is a constant pre-factor that accounts for the non-homogeneous flow profile around the NP. Note that the earlier analysis[15-17] implied that $\lambda = 0$ and the equations there do not contain λ, while we have introduced λ taking Supplementary Equation 11 into account. In addition, we may notice that in our experiments NPs contact the SLB via linkers, and accordingly λ contains a contribution by the linker length (above the SLB) but this contribution is nearly negligible.

Supplementary Note 3

Decomposition of 2D trajectories - For NPs under flow, we observed trajectories that showed a superposition of a random walk with a directed movement (Figure 1b in the main text). As expected, the drift occurs in direction of the flow (along the x-axis), while the movement perpendicular to the flow (y-axis) is expected to be purely random. This is further investigated in Supplementary Figure 1 showing a representative trajectory, its decomposition into x- or y-coordinates and the displacements along the x- or y-coordinates (calculated for data points that are separated by 2 frames: $\Delta x(i) = x(i+2) - x(i)$ and $\Delta y(i) = y(i+2) - y(i)$ with i denoting the frame number). For a pure 1D diffusion, *i.e.*, in the absence of directed movement, the average value of this coordinate difference should be zero, which is observed for Δy (Supplementary Figure 1e, blue line). Furthermore, the variance of Δy is equivalent to the mean squared displacement observed in y-direction and equals therefore $2 \cdot D_y \cdot \Delta t$ (with Δt denoting the lag time between 2 frames, $\Delta t = 2 \cdot \Delta t_0$, and D_y the diffusion coefficient in y-direction):

$$\mathrm{var}(\Delta y) = < \left(\Delta y - < \Delta y >\right)^2 > = < \Delta y^2 > = 2 \cdot D_y \cdot \Delta t \, . \qquad (13)$$

Hence, calculating the variance of Δy allows to directly extract D_y. The same holds for the x-direction with one difference: due to the directed movement, the average value of Δx is in theory now given by

$$< \Delta x > = v_x \cdot \Delta t \qquad (14)$$

and therefore non-zero (as observed for Δx; Supplementary Figure 1d, blue line). However, the variance of Δx is still proportional to the diffusion coefficient in the x-direction, D_x, despite the non-zero average value:

$$\mathrm{var}(\Delta x) = < (\Delta x - < \Delta x >)^2 > = 2 \cdot D_x \cdot \Delta t \,. \tag{15}$$

Hence, D_x and D_y can be independently extracted by calculating the variance of Δx and Δy, while taking the average value of Δx gives a convenient way to extract v_x from the trajectory. Further, as the SLB is a 2D isotropic medium, one expects that D_x and D_y should be equal, which is generally observed (see Supplementary Figure 2 for a representative example), if the experimental error is taken into account using

$$\frac{\sigma_{D_x}}{\sigma_{D_y}} = \sqrt{\frac{8}{3} \cdot \frac{N_p}{N - N_p}} \,. \qquad \text{(for } N_p << N\text{)} \tag{16}$$

This equation follows from the 1D version of Supplementary Equation 3

$$\frac{\sigma_{D_x}}{D_x} = \frac{\sigma_{D_y}}{D_y} = \sqrt{\frac{4}{3} \cdot \frac{N_p}{N - N_p}} \qquad \text{(for } N_p << N\text{)} \tag{17}$$

under the assumptions that random errors in the determination of v_x can be neglected (which can be achieved by choosing sufficiently high flow rates as described in Supplementary Note 1 “Accuracy of SPT-based size extraction”) and that the random errors in D_x and D_y are uncorrelated. As D_x and D_y are equal within experimental resolution (Supplementary Figure 2), it is apparent that the data extraction procedure successfully decouples the directed and the random particle movement. This further allows calculating the 2D diffusion coefficient D as arithmetic average of D_x and D_y, which further improves the accuracy in the quantification of the diffusion (compare Supplementary Equation 3 versus Supplementary Equation 17).

Supplementary Note 4

Calibration of 2D flow nanometry - As pointed out in the main text, the approach of 2D flow nanometry allows hydrodynamic shear forces F_s to be extracted from the stochastic (D) and deterministic components (v_x) of the flow-induced NP movement. The measured F_s can be translated into NP size based on Supplementary Equation 12 (or Equation 5 in the main text) provided that the unknown parameters A and λ have been determined. Although the parameter A has been derived in theory,[17] λ is not known *a priori* and conflicting values have been reported in the past.[8-14] Hence, it is necessary to determine A and λ using calibration experiments, which have been realized in this work using 3 independent batches of gold NPs that were well characterized using electron microscopy (size distribution given in Supplementary Figure 4, top row). Calibration of Supplementary Equation 12 was performed by (i) determination of the F_s distribution of each gold NP batch using 2D flow nanometry

(see Figure 2d), (ii) determination of the peak position in the size distributions determined using electron microscopy (after introducing a 5 nm shift accounting for the presence of a PEG corona surrounding the gold NPs; Supplementary Figure 4, top row), and finally (iii) fitting Supplementary Equation 12 to the peak values of the F_s and R distributions employing a weighted least squares fit. Since R, v_0 and η are known, only A and λ are fitting parameters, which were determined to be λ = 24.4 nm and $A\eta$ = 1 fN min µL^{-1} based on the data shown in Figure 2e. Note that attempts to fit Supplementary Equation 12 using λ = 0 failed to properly describe the data, indicating that λ cannot be neglected in our setting, further demonstrating the advantage of the calibration approach.

The quality of the calibration procedure was assessed by comparing the size distributions of the 3 gold NP batches obtained using 2D flow nanometry and electron microscopy (Supplementary Figure 4). Here, we take advantage of polydispersity of the gold NP samples, allowing to compare the results over a broader range. A good agreement was observed, with size distributions that are nearly identical in shape, width and peak position. The largest discrepancy in peak positions was observed for the largest gold NP batch, showing a systematic shift of only 7 nm, which is still a very good agreement in comparison to the overall width of the size distribution.

We continued assessing the quality of the calibration procedure by comparing size distributions of 3 different vesicle batches (Supplementary Figures 7 and 8) obtained with fluorescent NTA, DLS and 2D flow nanometry. Supplementary Figure 7 corresponds to 2 batches prepared using the extrusion (Supplementary Figure 7a) or sonication method (Supplementary Figure 7b) as described in the Methods section. Both batches showed a peak size around 37 nm but differed in their polydispersity (polydispersity index: PDI = 0.08 for extruded and 0.16 for sonicated vesicles as determined using DLS). Supplementary Figure 8 shows the results for an additional vesicle batch that has also been created by extrusion, but using a pore size of 100 nm in order to shift the size distribution to larger values. Interestingly, we observed that the size distributions obtained using 2D flow nanometry and fluorescent NTA were in good agreement for the right edge of the distribution, but deviations are observed at the left edge, corresponding to small vesicle sizes. Hence, 2D flow nanometry suggested the presence of small vesicles, in agreement with the DLS measurements (Supplementary Figure 7, right column), that were not observed in the NTA size distributions. This problem does not seem to be connected with resolution issues of NTA, as it was observed even for large vesicles (Supplementary Figure 8) that should be resolvable by NTA as judged by the results of the smaller vesicle batches (Supplementary Figure 7).

Despite these small deviations, the extracted size distributions showed good agreement in shape and peak position (*e.g.*, accessible from the corresponding cumulative distribution functions, Supplementary Figure 8b) further validating the calibration procedure. We therefore decided to add the vesicle data to the calibration plot (Figure 2e) in order to investigate the consistency of the data analysis

(Supplementary Figure 10, red circles). The vesicle data matched well to the result of the initial calibration procedure (Supplementary Figure 10, blue line). Moreover, adding these data points in the fitting procedure of Eq. 12 yielded λ = 30.4 nm and $A\eta$ = 0.9 fN min µL^{-1}, which barely deviated (Supplementary Figure 10, red line) from the initial result (Supplementary Figure 10, blue line).

Supplementary Note 5

Vesicle linking - Vesicles were linked to a SLB using cholesterol-modified DNA strands as described previously (Supplementary Figure 6).[18] DNA-strands were incorporated into the SLB by incubation of the SLB with a DNA (SLB DNA) suspension (concentration 12 nM; flow rate 20 µL min^{-1} for 20 min), followed by rinsing with the Tris-HCl buffer (flow rate 20 µL min^{-1} for 20 min). In parallel, 12.5 µL of fluorescently labeled vesicles (lipid concentration 1 mg mL^{-1}) were mixed with 8 µL of a DNA (vesicle DNA) suspension (concentration 50 nM), followed by further dilution with TRIS-HCl buffer to obtain a lipid concentration of 0.125 µg mL^{-1}. This suspension was injected into the PDMS microfluidic channel and tethering of the SUVs (by hybridization of the complementary strands) was followed in real time using TIRF and blocked by injecting TRIS-HCl buffer, once a feasible surface coverage was obtained (on the order of 2 vesicles per 100 µm^2, reached during incubation times of few 10 sec). Afterward, single particle tracking (SPT) movies of the linked vesicles were recorded.

This procedure prevents linker-induced aggregation of vesicles, since linking is achieved by hybridization of 2 different kinds of DNA-strands, each of them incorporated in either the vesicles or the SLB. Further, it has been shown that the double cholesterol-terminated DNA strands have high inserting efficiency and are stably bound to the bilayers on the timescale of the experiment,[19, 20] making DNA transfer between the SLB and the vesicles negligible.

Concerning the linker-induced vesicle aggregation, we may add that, in principle, the linkers themselves may tend to aggregate in the SLB and it might result in the vesicle aggregation. This mechanism implies aggregation of linkers belonging to different vesicles. Under our conditions, each vesicle has only a few linkers, and the aggregation of linkers belonging to different vesicles is energetically unfavourable because it may occur only if vesicles are appreciably deformed (this costs energy).

Supplementary Note 6

Limitations of 2D flow nanometry

Linking strategy – 2D flow nanometry uses the Einstein-Smoluchowski relation to determine the shear force acting on NPs that are linked to a SLB at the bottom of a microfluidic channel. This is achieved by monitoring NP diffusion coefficient and flow-induced velocity and therefore **requires a NP linking strategy that ensures a non-zero NP mobility after linking**. For vesicle-like biological NPs (*e.g.*, enveloped

virions, exosomes, liposomes etc.) this can be achieved using cholesterol-equipped DNA strands (as done in this study). Note that the insertion of cholesterol into gel-phase bilayers also takes easily place, and accordingly the lipid bilayer of the NPs can be in either fluid or gel phase, without limiting the applicability of cholesterol-mediate linking, making this linking strategy generically applicable to this NP class and very feasible. For NPs lacking a lipid shell it is necessary to find a suitable NP surface modification to enable linking to the SLB. This can easily be done because a large number of linking strategies has been developed in the past decades. Exemplarily, we used here streptavidin-biotin bonds to link gold NPs to biotinylated lipids in the SLB, but other strategies are applicable as well, since a variety of functionalized lipids are commercially available today.

Properties of the SLB – The SLB at the bottom of the microfluidic channel has to be of **high quality and in fluid phase** in order to provide sufficient NP mobility after linking. The exact composition is not important, since its main purpose is to provide a fluid interface that can be used to link and move NPs. Best results will be obtained using synthetic lipids, which often simplifiy SLB formation and result in excellent SLB fluidity. We additionally added PEGylated lipids to our SLB, which form cushion-like structures underneath the SLB, thereby decoupling motion within the SLB from surface effects and further improving SLB fluidity.

Size range of the NPs – The approach in its current version requires that the **NP diffusion is dominated by the linker**, *i.e.*, the friction coefficient of the linker within the SLB exceeds the friction coefficient of the NP within the solution. For single linker diffusion coefficients on the order of 1 $\mu m^2\ s^{-1}$, this constrain will be broken if R approaches or exceeds 200 nm. In this case, the velocity of flow-induced directed movement will become comparable or equal to the fluid velocity at the NP's midplane, *i.e.*, the NP velocity becomes comparable to the velocity of the surrounding flow, causing Supplementary Equation 12 to break down. This upper size limit can be extented by using firmer linking that decreases the NP diffusion coefficient after linking far below 1 $\mu m^2\ s^{-1}$. To achieve *e.g.* 0.1 $\mu m^2\ s^{-1}$ using cholesterol-mediated linking, the NPs would have to be linked via approximately 10 DNA-linkers, which is experimentally accessible.[24] Moreover, it should be noted that smaller NPs require larger flow rates to be precisely characterized. Increasing the flow rate above a certain threshold (approximately 100 $\mu L\ min^{-1}$ in our settings) induces a directed movement of the SLB, also causing Supplementary Equation 12 to break down. Based on Figure 2e such large flow rates would be required for NP sizes on the nm scale, which is comparable to the size of the linkers used in this study. We therefore conclude that the **applicable size range** of 2D flow nanometry (in its current version) is given by **20 – 400 nm** (in diameter), although usage of shorter linkers should allow smaller NPs to be characterized and the upper size limit should be extendable using firmer linking.

Aggregated NPs – The approach was **developed to characterize isolated NPs** and its extension toward aggregated NPs is not obvious. The NPs of the latter category can also hardly be characterized using complementary approaches. The shape of aggregated NP complexes will usually deviate from a sphere, which is a key assumption in NTA, DLS and 2D flow nanometry and hence all these techniques are expected to report a kind of averaged hydrodynamic radius, *i.e.*, a value between the smallest and largest extension of the NP complex.

Supplementary Note 7

Flow-induced vesicle deformation - In principle, the flow may induce deformation of attached vesicles, and this is turn may modify the F_s distribution depending on the flow rate, *i.e.*, different distributions would be observed for different flow rates. This was, however, not observed in our experiments for vesicle diameter up to 150 nm (Supplementary Figure 11), indicating absence of flow-induced vesicle deformation for a diameter below 150 nm.

To clarify whether the flow-induced vesicle deformation is likely in our case, we can also use the theory and complementary experimental data. Following this line, we note that the scale of the vesicle bending energy is $8\pi\kappa$; where κ is the bending constant [see *e.g.* Eq. (2.54) in Ref. 21], and accordingly the scale of the force needed to appreciably deform a vesicle is $8\pi\kappa/R$. For relatively small vesicles like those employed in our study, the recent experiments indicate that the scale of κ is $(1-2)\times10^{-19}$ J.[22, 23] Using these values in combination with R = 50, we obtain that the deformation is appreciable if the force is comparable with or larger than $(0.5 - 1) \times 10^{-12}$ N. In our experiment, the scale of the force, $(1 - 2) \times 10^{-14}$ N (see, *e.g.*, Supplementary Figure 5), is much smaller. Thus, flow-induced vesicle deformation is unlikely.

Supplementary References

1. Johansson Fast, B. *PhD thesis*, Chalmers University of Technology, (2016).
2. Larsen, J., Hatzakis, N.S. & Stamou, D. Observation of inhomogeneity in the lipid composition of individual nanoscale liposomes. *JACS*, **133**, 10685-10687, (2011).
3. Einstein, A. Über die von der molekularkinetischen Theorie der Wärme geforderte Bewegung von in ruhenden Flüssigkeiten suspendierten Teilchen. *Annalen der Physik (Berlin)*, **322**, 549-560, (1905).
4. Qian, H., Sheetz, M.P. & Elson, E.L. Single particle tracking. Analysis of diffusion and flow in two-dimensional systems. *Biophysical Journal*, **60**, 910-921, (1991).
5. Saxton, M. Single-particle tracking: The distribution of diffusion coefficients. *Biophysical Journal*, **72**, 1744-1753, (1997).
6. Michalet, X. & Berglund, A.J. Optimal diffusion coefficient estimation in single-particle tracking. *Physical Review E*, **85**, 061916, (2012).
7. Bortz, J. Statistik: für Sozialwissenschaftler, Edn. Fünfte, vollständig überarbeitete und aktualisierte Auflage. (Springer Berlin Heidelberg, 1999).
8. Voinova, M.V., Rodahl, M., Jonson, M. & Kasemo, B. Viscoelastic acoustic response of layered polymer films at fluid-solid interfaces: Continuum mechanics approach. *Physica Scripta*, **59**, 391-396, (1999).
9. Kazoe, Y. & Yoda, M. Measurements of the near-wall hindered diffusion of colloidal particles in the presence of an electric field. *Applied Physics Letters*, **99**, 124104, (2011).
10. Choi, C., Westin, K. & Breuer, K. Apparent slip flows in hydrophilic and hydrophobic microchannels. *Physics of Fluids*, **15**, 2897-2902, (2003).
11. Cross, B., Steinberger, A., Cottin-Bizonne, C., Rieu, J. & Charlaix, E. Boundary flow of water on supported phospholipid films. *Europhysics Letters*, **73**, 390-395, (2006).
12. Joseph, P. & Tabeling, P. Direct measurement of the apparent slip length. *Physical Review E*, **71**, 035303, (2005).
13. Leroy, S., Steinberger, A., Cottin-Bizonne, C., Trunfio-Sfarghiu, A. & Charlaix, E. Probing biolubrication with a nanoscale flow. *Soft Matter*, **5**, 4997-5002, (2009).
14. Bonaccurso, E., Kappl, M. & Butt, H.J. Hydrodynamic force measurements: boundary slip of water on hydrophilic surfaces and electrokinetic effects. *Physical Review Letters*, **88**, 076103, (2002).
15. Goldman, A., Cox, R. & Brenner, H. Slow viscous motion of a sphere parallel to a plane wall—II Couette flow. *Chemical Engineering Science*, **22**, 653-660, (1967).
16. Jönsson, P., Gunnarsson, A. & Höök, F. Accumulation and separation of membrane-bound proteins using hydrodynamic forces. *Analytical Chemistry*, **83**, 604-611, (2011).
17. Jönsson, P. & Jönsson, B. Hydrodynamic forces on macromolecules protruding from lipid bilayers due to external liquid flows. *Langmuir*, **31**, 12708-12718, (2015).
18. Benkoski, J. & Höök, F. Lateral mobility of tethered vesicle - DNA assemblies. *Journal of Physical Chemistry B*, **109**, 9773-9779, (2005).
19. Pfeiffer, I. & Höök, F. Quantification of oligonucleotide modifications of small unilamellar lipid vesicles. *Analytical Chemistry*, **78**, 7493-7498, (2006).
20. Pfeiffer, I. & Höök, F. Bivalent cholesterol-based coupling of oligonucletides to lipid membrane assemblies. *JACS*, **126**, 10224-10225, (2004).
22. Reviakine, I., Gallego, M., Johannsmann, D. & Tellechea, E. Adsorbed liposome deformation studied with quartz crystal microbalance. *Journal of Chemical Physics* **136**, 084702, (2012).

23. Takechi-Haraya, Y., Sakai-Kato, K., Abe, Y., Kawanishi, T., Okuda, H. & Goda, Y. Atomic force microscopic analysis of the effect of lipid composition on liposome membrane rigidity. *Langmuir* **32**, 6074-6082, (2016).
24. Block, S., Zhdanov, V.P. & Höök, F. Quantification of Multivalent Interactions by Tracking Single Biological Nanoparticle Mobility on a Lipid Membrane. *Nano Letters* **16**, 4382-4390, (2016).
